# Image Fusion Method Based on Snake Visual Imaging Mechanism and PCNN

**DOI:** 10.3390/s24103077

**Published:** 2024-05-12

**Authors:** Qiang Wang, Xuezhi Yan, Wenjie Xie, Yong Wang

**Affiliations:** College of Communication Engineering, Jilin University, Changchun 130012, China; wq17122824802022@163.com (Q.W.); yanxz@jlu.edu.cn (X.Y.); xiewj98@163.com (W.X.)

**Keywords:** rattlesnake, visible image, infrared image, image fusion, PCNN

## Abstract

The process of image fusion is the process of enriching an image and improving the image’s quality, so as to facilitate the subsequent image processing and analysis. With the increasing importance of image fusion technology, the fusion of infrared and visible images has received extensive attention. In today’s deep learning environment, deep learning is widely used in the field of image fusion. However, in some applications, it is not possible to obtain a large amount of training data. Because some special organs of snakes can receive and process infrared information and visible information, the fusion method of infrared and visible light to simulate the visual mechanism of snakes came into being. Therefore, this paper takes into account the perspective of visual bionics to achieve image fusion; such methods do not need to obtain a significant amount of training data. However, most of the fusion methods for simulating snakes face the problem of unclear details, so this paper combines this method with a pulse coupled neural network (PCNN). By studying two receptive field models of retinal nerve cells, six dual-mode cell imaging mechanisms of rattlesnakes and their mathematical models and the PCNN model, an improved fusion method of infrared and visible images was proposed. For the proposed fusion method, eleven groups of source images were used, and three non-reference image quality evaluation indexes were compared with seven other fusion methods. The experimental results show that the improved algorithm proposed in this paper is better overall than the comparison method for the three evaluation indexes.

## 1. Introduction

Image fusion is a process of using different sensors to generate richer and higher quality images from the same scene through computer technology. Image fusion processing can extract the effective information and available complementary information in the image, filter the redundant information and invalid information in the source image, generate a robust or informative image, and improve the image quality.

Image fusion has been studied for more than 40 years and has been widely used. Researchers have improved fusion methods from various angles. Among them, the fusion of visible image and infrared image has been paid more attention. Fusion algorithms based on traditional image processing methods are constantly evolving, including many different traditional methods, such as the multi-scale transform fusion algorithm [1,2], sparse representation image fusion algorithm [3] and some other traditional methods [4,5]. This traditional method is generally stable, no training is needed and the fusion effect is also very good, but it is generally complex to use in calculations and low in operation efficiency. There may be some problems in the processing of details. Nowadays, fusion algorithms based on deep learning methods are becoming more and more popular, including methods based on convolutional neural networks [6,7], methods based on generative adversarial networks [8], methods based on autoencoder networks [9] and so on. This method generally provides a good fusion effect and rich details, but requires a large amount of data for training. However, in some applications, a large amount of training data cannot be obtained.

The research findings in the field of biology provide a new idea for the study of image fusion methods. Researchers have studied the biological mechanisms of visual perception and infrared vision in snakes. In 1953, by dissecting the optic nerve fibers of frogs, Kuffler [10] discovered the activity of ganglion cells and the existence of two basic receptor types based ON various firing patterns in the retina, ON/OFF centers surrounding cells. Hodgkin and Huxley [11] proposed the passive membrane equation through scientific research to describe center-surround shunting neural networks (CSSNNs). In 1978, Hartline et al. [12] studied the visual function and infrared perception of ventral subfamily snakes (rattlesnakes) using electrophysiological methods, and pointed out that visible light and infrared sensing neurons were distributed in the optic tectum of these snakes, and clarified the existence of bihump cells. In 1981, Newman and Hartline [13] discovered that the two-mode cells of rattlesnakes can receive and process information from both infrared and visible light, and automatically fuse infrared and visible images naturally. Chen et al. then temporarily blocked some sensors of the pit viper, demonstrating that infrared and visible information complement each other for the pit viper to hunt prey and inhibit each other in the localization process [14].

On the basis of this research on the infrared sensing organs of snakes, some researchers have proposed some novel fusion methods of infrared and visible light from the perspective of mimicking the visual imaging mechanism of snakes. In 1997, Waxman et al. [15] imitated the physiological mechanism of rattlesnakes and proposed an adversarial fusion method of night vision images and infrared images. But the pseudo-color image generated by this method is distorted and has low visibility, which is not conducive to human observation. Reinhard et al. [16] proposed a method for color transfer between two color images. Li [17] and Zhang et al. [18] have improved the classical receptive field model of snakes and achieved good results.

The pulse-coupled neural network model is the third generation of untrained artificial neural networks, which is different from the traditional artificial neural network. It is inspired by mammals. In 1990, Eckhorn et al. [19] proposed a neural network model based on signal transduction of neurons in the cat visual cortex. In 1999, Johnson and Padgett [20] modified this model for image processing and named it the PCNN. Through linear addition and modulation coupling, the PCNN reflects exponential attenuation and time delay of bioelectrical transmission. So, the PCNN has better processing ability for adjacent excitation signals and can be used for image fusion, image segmentation, etc. The PCNN does not require training.

This paper is based on the research of the image fusion method of the rattlesnake vision imaging system. From the perspective of bionics, the fusion method of infrared images and visible images is designed to simulate the fusion mechanism of infrared signals and visible signals of rattlesnakes. Different from deep learning methods, this kind of method does not require a large amount of training data, and can still achieve fusion in some applications where a large amount of training data cannot be obtained. So far, all the fusion methods used to simulate snakes generate pseudo-color images because human eyes can recognize objects faster in color images than in gray ones. However, the pseudo-color images generated by these methods may make the details unclear. Therefore, when building a model simulating the vision mechanism of snakes, this paper does not use the step of directly mapping the processed images to RGB three-channel to generate pseudo-color images, but combines the model with the PCNN network.

## 2. Related Work

### 2.1. Visual Receptive Field and Mathematical Mode

#### 2.1.1. Visual Receptive Field

In the 1930s, Hartline became the first person to document the axonal ganglion cells of a single retina by dissecting optic nerve fibers from frogs. He identified three types of retinal ganglion cells: ON cells that fire strongly when the retina is illuminated, OFF cells that fire when light is turned OFF, and ON/OFF cells that react briefly to both the turning on and turning off of light. He proved that each cell was sensitive to only a small illuminated area on the surface of the retina, which he called the receptive field of the cell. Kuffler then discovered in cats that the receptive field of each ganglion cell is actually composed of two concentrically arranged regions: an excitatory central region and an antagonistic surround region. Stimulating the central area with a small spot will cause a strong response, while a larger spot stimulus will produce a diminished response (antagonization) when it spreads to the surrounding area. When the irradiation range of light spot is limited to the center of its receptive field, ON/OFF cells have a strong instantaneous response to both the beginning and the end of light. When ON cells are stimulated by light intensity or local light enhancement, the frequency of the nerve pulse is increased. The OFF cells are activated when the light intensity is removed or when the local light intensity is reduced, and the frequency of their nerve pulse is increased. When the size of the spot increases to the surrounding area, the response to light increment and light decrement is decreased, proving that the surrounding area causes the opposite response.

**Figure 1:**
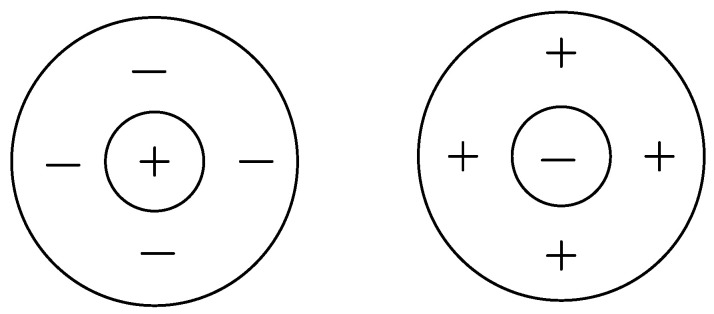
ON/OFF central receptive field model (left is ON receptive field; right is OFF receptive field).

According to the study of anatomy and physiology, the common receptive field of retinal nerve cells can be divided into ON-center and OFF-center ganglion cells. The ON ganglion cells are located in the ON excitatory region and surrounded by the OFF inhibitory region. The OFF ganglion cells are located in the OFF excitatory region and surrounded by the ON inhibitory region. In Figure 1, “+” represents the excitatory region and “−” represents the inhibitory region.

#### 2.1.2. ON/OFF—Mathematical Model of Central Receptive Field

At first, A.F.Huxley et al. proposed the passive membrane equation to simulate the exchange of cell membrane ion currents in physiology. Later, Newman et al. [21] used Grossberg’s centered surround shunt neural network to build an image fusion model based on snakes. The formula is as follows:

(1) $$\frac{d}{dt}X(x,y)=-A[X(x,y)-D]+[E-X(x,y)]C(x,y)-[F+X(x,y)]S(x,y)$$

(2) $$\frac{d}{dt}Y(x,y)=-A[Y(x,y)-\bar{D}]+[E-Y(x,y)]S(x,y)-[F+Y(x,y)]C(x,y)$$

where $X\left(x,y\right)$ and $Y\left(x,y\right)$ are the ON center versus cell response and OFF center versus cell response, respectively. A represents the attenuation constant of the cell, D and $\bar{D}$ represent the basal activity of ON and OFF against cells, respectively, E and F are the polarization constants, $C\left(x,y\right)$ and $S\left(x,y\right)$ represent the central region and surrounding region of the receptive field obeying the Gaussian distribution, respectively, and the formula is as follows:

(3) $$C(x,y)=I(x,y)*W_{c}(x,y)=\frac{1}{2\pi \sigma_{c}^{2}}\sum_{m,n}I(x-m,y-n)exp(-\frac{m^{2}+n^{2}}{2\sigma_{c}^{2}})$$

(4) $$S(x,y)=I(x,y)*W_{s}(x,y)=\frac{1}{2\pi \sigma_{s}^{2}}\sum_{p,q}I(x-p,y-q)exp(-\frac{p^{2}+q^{2}}{2\sigma_{s}^{2}})$$

The above formulas describe the instantaneous changes in cells after stimulation. When the cell response eventually tends to balance, the following equation is obtained.

ON against cell output:

(5) $$X(x,y)=\frac{AD+EC(x,y)-FS(x,y)}{A+C(x,y)+S(x,y)}$$

OFF against cell output:

(6) $$Y(x,y)=\frac{AD+ES(x,y)-FC(x,y)}{A+C(x,y)+S(x,y)}$$

### 2.2. Six Dual-Mode Cell Fusion Mechanisms and Mathematical Models in Rattlesnakes

ON, OFF, and ON/OFF cells in the retina and their antagonistic center-surrounding tissue form the basic structure of all vertebrate visual systems, and spatial antagonism is common in the cellular receptive field of the visual system. A great number of dual-mode cells exist in the optic tectum of venomous snakes such as pythons and rattlesnakes. These cells have different nonlinear responses when receiving infrared and visible light stimulation. These responses are roughly divided into six categories, and the following will be briefly introduced to these six responses.

#### 2.2.1. “OR” Cells

When the “OR” cell receives two kinds of stimulus signals, the visible light signal and infrared signal, it can not only respond to any single one of the two kinds of stimulus signals, but also respond to two kinds of stimulus signals that exist at the same time, and it will result in a gain effect when both signals are present at the same time and stimulate the cell. Therefore, the weighted method is adopted to simulate the physiological mechanism of “OR” cells.

When $I_{V}(x,y)<I_{IR}(x,y)$, the mathematical model is

(7) $$I_{OR}(x,y)=nI_{V}(x,y)+mI_{IR}(x,y)$$

When $I_{V}(x,y)>I_{IR}(x,y)$, the mathematical model is

(8) $$I_{OR}(x,y)=mI_{V}(x,y)+nI_{IR}(x,y)$$

where m > 0.5, n < 0.5, $I_{OR}\left(x,y\right)$ represents the image obtained after the processing of the “OR” cell mathematical model.

#### 2.2.2. “AND” Cells

When the “AND” cell receives two kinds of stimulus signals, the visible light signal and infrared signal, it can only produce an obvious response when the two kinds of stimulus signals are present at the same time. And when either of the two kinds of stimulus signals separately stimulate the cell, there is basically no response or only a weak response. Therefore, the weighted method is adopted to simulate the physiological mechanism of “AND” cells.

When $I_{V}(x,y)<I_{IR}(x,y)$, the mathematical model is

(9) $$I_{AND}(x,y)=mI_{V}(x,y)+nI_{IR}(x,y)$$

When $I_{V}(x,y)>I_{IR}(x,y)$, the mathematical model is

(10) $$I_{AND}(x,y)=nI_{V}(x,y)+mI_{IR}(x,y)$$

where m > 0.5, n < 0.5, $I_{AND}\left(x,y\right)$ represents the image obtained after the processing of “AND” cell mathematical model.

#### 2.2.3. Enhanced Cell Mathematical Model

(1) The mathematical model of infrared enhanced visible light: When the cell receives two kinds of stimulus signals, the visible light signal and infrared signal, a response is generated only when the visible light signal separately stimulates the cell, and when the infrared signal separately stimulates the cell, there is basically no response or only a weak response. However, when two kinds of signals stimulate the cell at the same time, the response generated will be enhanced. So, the infrared signal plays a role in enhancing the response in the cell.

When the received two signals stimulate the cell simultaneously, the response generated by the visible light signal stimulation of the cell is the most significant part, while the stimulation of the infrared signal to the cell enhances the response generated by the visible light signal stimulation. Therefore, visible light signal plays a dominant role in the establishment of mathematical models to simulate the physiological mechanism of this cell, while the infrared signal uses the exponential function to represent the enhancement effect. The mathematical model is

(11) $$I_{IR+V}(x,y)=I_{V}(x,y)exp[I_{IR}(x,y)]$$

(2) The mathematical model of visible enhanced infrared light: When the cell receives two kinds of stimulus signals, the visible light signal and infrared signal, the response is generated only when the infrared signal stimulates the cell alone, and basically no response or only a weak response is generated when the visible light signal stimulates the cell alone. However, when the two kinds of signals stimulate the cell at the same time, the response generated by the cell will be enhanced. So, visible light signal plays a role in assisting enhancement in this cell.

Therefore, in the establishment of mathematical models to simulate the physiological mechanism of this cell, the infrared signal plays a dominant role, while the visible signal uses the exponential function to represent the enhancement effect. The mathematical model is

(12) $$I_{V+IR}(x,y)=I_{IR}(x,y)exp[I_{V}(x,y)]$$

#### 2.2.4. Inhibited Cell Mathematical Model

(1) The mathematical model of infrared suppression of visible light: When the cell receives two kinds of stimulus signals, the visible light signal and infrared signal, the response is generated only when the visible light signal stimulates the cell alone, while when the infrared signal stimulates the cells alone, there is basically no response or only a weak response. However, when the two kinds of signals stimulate the cell at the same time, the response produced by the cell will be weakened. Therefore, the infrared signal plays a role in inhibiting the response in the cell.

When the received two signals stimulate the cell simultaneously, the response generated by the visible light signal stimulation of the cell is the most significant part, while the stimulation of the infrared signal to the cell weakens the response generated by the visible light signal stimulation. Therefore, the visible light signal plays a dominant role in the establishment of mathematical models to simulate the physiological mechanism of this cell, while the infrared signal uses the logarithmic function to represent the inhibition effect. The mathematical model is as follows:

(13) $$I_{IR-V}(x,y)=I_{V}(x,y)log[I_{IR}(x,y)+1]$$

(2) The mathematical model of visible suppression of infrared light: When the cell receives two kinds of stimulus signals, the visible light signal and infrared signal, the response is generated only when the infrared signal stimulates the cell alone, and when the visible light signal stimulates the cell alone, there is basically no response or only a weak response. However, when the two kinds of signals stimulate the cell at the same time, the response produced by the cell will be weakened. Therefore, the visible light signal plays a role in inhibiting the response in this cell.

Therefore, in the establishment of mathematical models to simulate the physiological mechanism of this cell, the infrared signal plays a dominant role, while the visible signal uses the logarithmic function to represent the inhibition effect. The mathematical model is as follows:

(14) $$I_{V-IR}(x,y)=I_{IR}(x,y)log[I_{V}(x,y)+1]$$

### 2.3. Basic Theory of Pulse-Coupled Neural Networks

As a famous third-generation artificial neural network, the PCNN has its own advantages compared with other image processing methods. First, the PCNN model is derived from studies of the cat visual cortex. Its information processing is closer to human visual processing. And the PCNN has a flexible structure. In addition, the existing PCNN method also shows that the PCNN has a wide range of applications in image processing fields such as image fusion and image enhancement. Therefore, recently, the image fusion method based on PCNNs has attracted more attention from many experts because of its characteristics in the field of biology.

This section mainly introduces PCNN neuron model, simplified neuron model, and the operating mechanism of PCNN. Firstly, the standard model of PCNN and its simplified model are introduced.

**Figure 2:**
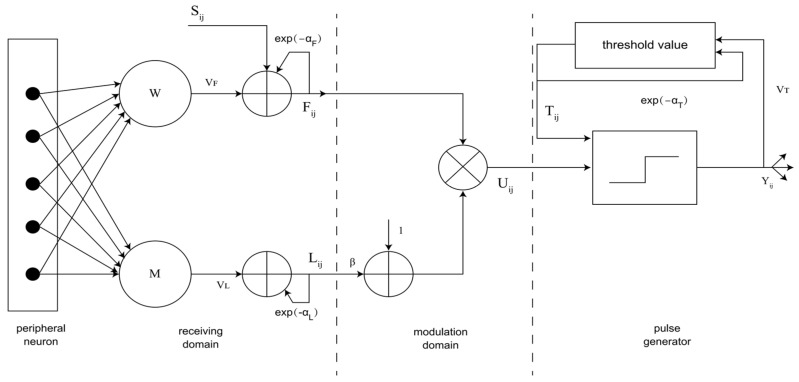
PCNN model (F_ij_: feed input, L_ij_: link input, S_ij_: external stimulus, M and W: connection weight matrix, α_L_ and α_F_: time attenuation constant, V_F_ and V_L_: inherent potential constant, U_ij_: internal state of the neuron, β: connection coefficient of the modulation domain, T_ij_: dynamic threshold, α_T_: time attenuation constant; V_T_: the threshold of firing neurons) [22].

The structure of PCNN neurons is shown in Figure 2. The neuron consists of an input part, a connection part and an impulse generator. Neurons receive input signals from feed inputs and link inputs. The feed input is the main input from the receiving area of the neuron. The receiving area of the neuron consists of adjacent pixels of the corresponding pixel in the input image. Link inputs are secondary inputs that are laterally connected to adjacent neurons. The difference between these inputs is that the feed input has a slower characteristic response time constant than the link input. The standard PCNN model is represented by the following formula. 

The role of the receive field is to receive the following two types of input:

(15) $$F_{ij}(n)=exp(-\alpha_{F})F_{ij}(n-1)+V_{F}\sum_{kl}M_{ijkl}Y_{kl}(n-1)+S_{ij}$$

(16) $$L_{ij}(n)=exp(-\alpha_{L})L_{ij}(n-1)+V_{L}\sum_{kl}W_{ijkl}Y_{kl}(n-1)$$

where $F_{ij}$ and $L_{ij}$ represent the feed input and the link input, respectively, $S_{ij}$ represents the external stimulus and $M_{ijkl}$ and $W_{ijkl}$ represent the connection weight matrix, which can regulate the influence of each neuron in the neighborhood of the central neuron. $\alpha_{L}$ and $\alpha_{F}$ represent the time attenuation constant, which determines the attenuation speed of channel F and channel L. Usually, $\alpha_{L}>\alpha_{F}$. $V_{F}$ and $V_{L}$ are the inherent potential constants, respectively, are the amplitude coefficients of the feed input and the connection input, and represent the amplitude adjustment constants of the connection domain. The energy transferred by the ignition neuron in the neighborhood to the central neuron can be scaled. The subscript ij indicates the position of the center pixel of the PCNN. The subscript kl represents the position of the adjacent pixel corresponding to the center pixel.

In the modulation domain, the following equation can be used:

(17) $$U_{ij}(n)=F_{ij}(n)[1+\beta L_{ij}(n)]$$

where $U_{ij}$ represents the internal state of the neuron and β represents the connection coefficient of the modulation domain, which can change the weight of the linked channels in the internal activity, and the value of β usually depends on different needs. If the influence from the L channel is expected to be large, β should be given a larger value. All neurons usually have the same value. But it is not absolute. Each neuron can have its own value.

The function of the pulse generator is to generate pulse output, which is composed of a threshold regulator, comparator and pulse generator, as shown below:

(18) $$T_{ij}(n)=exp(-\alpha_{T})T_{ij}(n-1)+V_{T}Y_{ij}(n)$$

(19) $$Y_{ij}(n)=\left\{\begin{array}{c} 1,\;U_{ij}(n)>T_{ij}(n)\\ 0,\;U_{ij}(n)\leq T_{ij}(n) \end{array}\right.$$

where $T_{ij}(n)$ represents the dynamic threshold and $\alpha_{T}$ represents the time attenuation constant, and the rate at which the threshold decays in the iterative process. It directly determines the firing time of neurons and is an important parameter. Smaller $\alpha_{T}$ can make the PCNN work more intricate, but it takes a significant amount of time to complete the processing. Larger $\alpha_{T}$ values can reduce the running time of PCNNs. $V_{T}$ determines the threshold of firing neurons, which is usually constant. When the internal state $U_{ij}$ of the neuron is greater than the threshold value $T_{ij}$, that is, the neuron meets the condition $U_{ij}(n)>T_{ij}(n)$, the neuron will generate a pulse, also known as firing once.

Since the formula of the standard model of PCNNs is too complicated, a simplified model of PCNNs is proposed later with the improvement of research. The formula of this simplified model is an improvement of Equation (15) and is shown below:

(20) $$F_{ij}(n)=S_{ij}$$

## 3. Fusion Method Based on Rattlesnake Imaging Mechanism and PCNN

In the previous chapter, according to the physiological mechanism of rattlesnake double-mode cells, six cell types were classified, and the mechanism and mathematical model of these six cells were introduced. The basic theory of the PCNN model was also briefly introduced. An improved algorithm based on the rattlesnake imaging mechanism and PCNN is proposed to solve the problem of detail loss in fused images.

**Figure 3:**
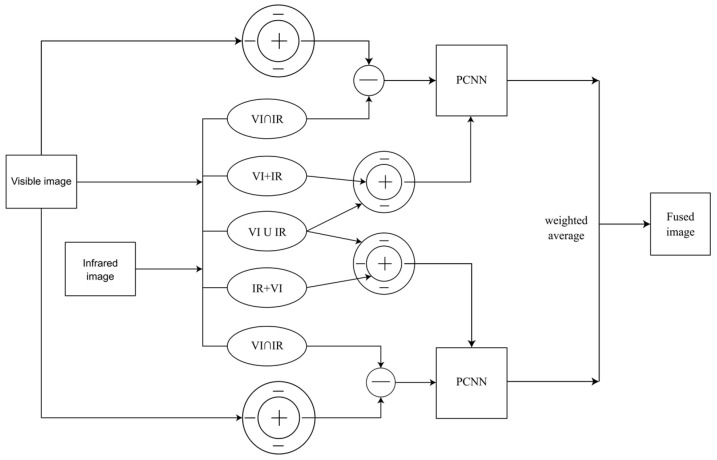
Model structure based on rattlesnake dual-mode mechanism and PCNN.

The structure of the improved algorithm is shown in Figure 3. Infrared and visible light source images are denoted as IR and VI.

First, VI is input into the ON confrontation system. According to the characteristics of the ON-centered receptive field, the input VI is enhanced to obtain the enhanced visible image VI_ON.

Second, VI and IR are treated with “OR”, “AND” and “enhancement” according to the simulated mathematical model by simulating the six dual-mode cell working mechanisms of rattlesnake vision, and the information of VI∩IR, VI∪IR, VI+IR (visible light enhanced infrared) and IR+VI (infrared enhanced visible light) is obtained, respectively.

Third, after subtracting the common information (VI∩IR) obtained by “AND” cells from the enhanced visible light image VI_ON, the unique information vi of the enhanced visible light is obtained.

Fourth, VI∪IR input surrounds the suppression area and corresponds to the suppression signal; VI+IR input to the central excitation area corresponds to the excitation signal, and the final output is OR+VI_IR. At the same time, VI∪IR input surrounds the suppression area, corresponding to the suppression signal; IR+VI input surrounds the central excitation area, corresponding to the excitation signal, and the final output is OR+IR_VI. That is, VI+IR, IR+VI and VI∪IR are input into two ON confrontation systems at the same time.

Then, OR+VI_IR and vi are entered into a PCNN, resulting in PCNN1. At the same time, OR+IR_VI and vi are entered into another PCNN to obtain PCNN2.

Finally, the two-image information of PCNN1 and PCNN2 is weighted to obtain the final fusion image.

## 4. Results and Discussion

Following on from the above discussion, this section will conduct experimental simulation for the proposed improved algorithm and compare it with other fusion methods. The image fusion effect of the improved algorithm is compared from two aspects, subjective evaluation and objective evaluation.

To observe the image fusion effect of our improved algorithm more directly and concretely, seven fusion algorithms are prepared in this section as comparative experiments. In this paper, we briefly introduce the following methods to compare with the improved algorithm.

First, the improved fusion algorithm proposed by Li [17] is taken as one of the comparative experiments. In order to facilitate the identification of comparison methods in subsequent evaluation, it is denoted as Li here.

Second, the improved MIT color fusion algorithm proposed by Zhang et al. [18] is taken as one of the comparative experiments. In order to facilitate the identification of comparison methods in subsequent evaluation, it is denoted as Zhang here.

Third, the improved image fusion model based on rattlesnake double-mode cells proposed by Wang et al. [23] is taken as one of the comparative experiments. In order to facilitate the identification of comparison methods in subsequent evaluation, it is denoted as Wang here.

Fourth, the experiment based on Gradient Transfer Fusion and total change (TV) proposed by Ma et al. [24] is taken as one of the comparative experiments. In order to facilitate the identification of comparison methods in subsequent evaluation, it is denoted as GTF here.

Fifth, the fusion method of latent low-rank representation proposed by Li et al. [25] is taken as one of the comparative experiments. In order to facilitate the identification of comparison methods in subsequent evaluation, it is denoted LatLRR here.

Sixth, the image fusion method based on multi-resolution singular value decomposition proposed by Naidu et al. [26] is taken as one of the comparative experiments. In order to facilitate the identification of comparison methods in subsequent evaluation, it is denoted as MSVD here.

Seventh, the multi-sensor image fusion method based on the fourth-order partial differential equations proposed by Bavirisetti et al. [27] is taken as one of the comparative experiments. In order to facilitate the identification of comparison methods in subsequent evaluation, it is denoted as FPDE here.

Eighth, in order to facilitate the identification of the comparative methods in subsequent evaluation, the proposed improved algorithm is recorded as Our.

### 4.1. Subjective Evaluation

Aiming at the improved method Our proposed in the previous section and other fusion methods compared with Our, 11 groups of source images of different scenes are used in this section for comparative simulation. Among them, seven groups of source images are from the TNO dataset [28] and four groups of source images are from the MSRS (Multi-Spectral Road Scenarios for Practical Infrared and Visible Image Fusion) dataset. The experimental parameters of Our are as follows: $\sigma_{s}$ = 500, $\sigma_{c}$ = 2.83, A = 1, D = 0, E = 900 and F = 1. In this section, a comparative analysis is made from the subjective evaluation, namely the visual effect of the fused image observed by human eyes.

#### 4.1.1. TNO Dataset

**Figure 4:**
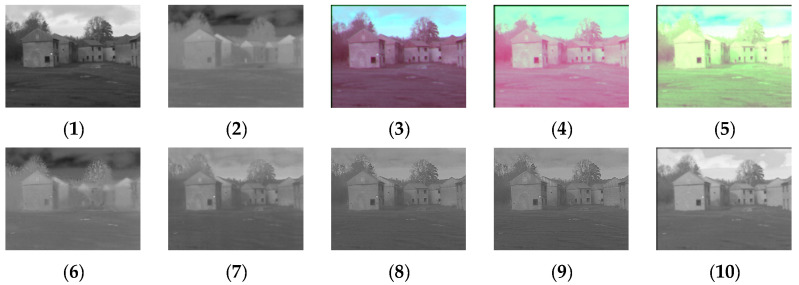
Comparison of experimental results of the first set of graphs in the TNO dataset. (**1**) visible image; (**2**) infrared image; (**3**) Li [17]; (**4**) Zhang [18]; (**5**) Wang [23]; (**6**) GTF [24]; (**7**) LatLRR [25]; (**8**) MSVD [26]; (**9**) FPDE [27]; (**10**) Our.

**Figure 5:**
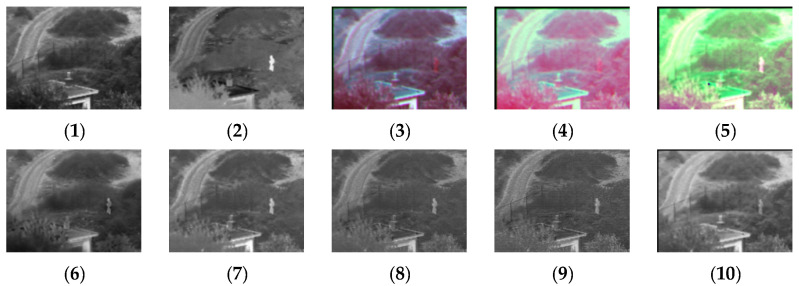
Comparison of experimental results of the second set of graphs in the TNO dataset. (**1**) visible image; (**2**) infrared image; (**3**) Li [17]; (**4**) Zhang [18]; (**5**) Wang [23]; (**6**) GTF [24]; (**7**) LatLRR [25]; (**8**) MSVD [26]; (**9**) FPDE [27]; (**10**) Our.

**Figure 6:**
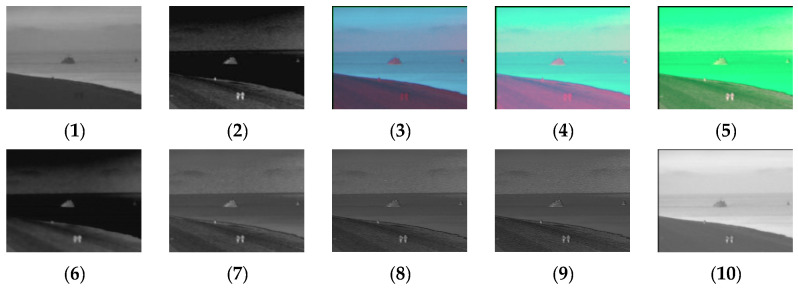
Comparison of experimental results of the third set of graphs in the TNO dataset. (**1**) visible image; (**2**) infrared image; (**3**) Li [17]; (**4**) Zhang [18]; (**5**) Wang [23]; (**6**) GTF [24]; (**7**) LatLRR [25]; (**8**) MSVD [26]; (**9**) FPDE [27]; (**10**) Our.

**Figure 7:**
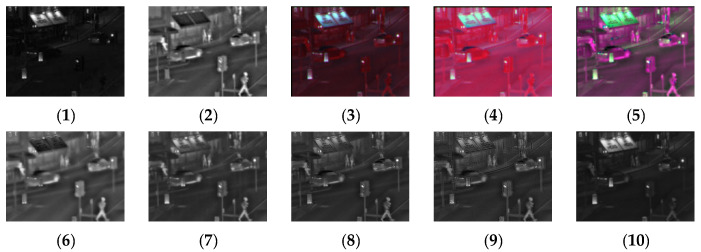
Comparison of experimental results of the fourth set of graphs in the TNO dataset. (**1**) visible image; (**2**) infrared image; (**3**) Li [17]; (**4**) Zhang [18]; (**5**) Wang [23]; (**6**) GTF [24]; (**7**) LatLRR [25]; (**8**) MSVD [26]; (**9**) FPDE [27]; (**10**) Our.

**Figure 8:**
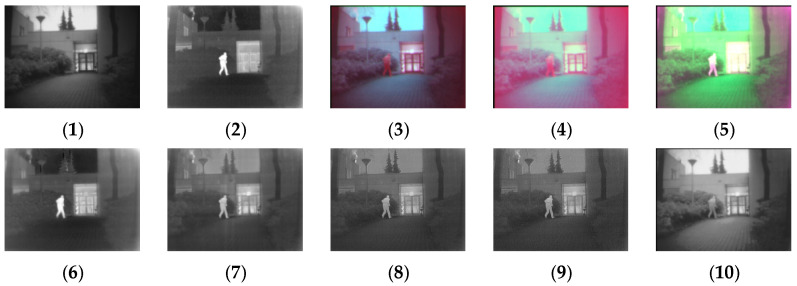
Comparison of experimental results of the fifth set of graphs in the TNO dataset. (**1**) visible image; (**2**) infrared image; (**3**) Li [17]; (**4**) Zhang [18]; (**5**) Wang [23]; (**6**) GTF [24]; (**7**) LatLRR [25]; (**8**) MSVD [26]; (**9**) FPDE [27]; (**10**) Our.

**Figure 9:**
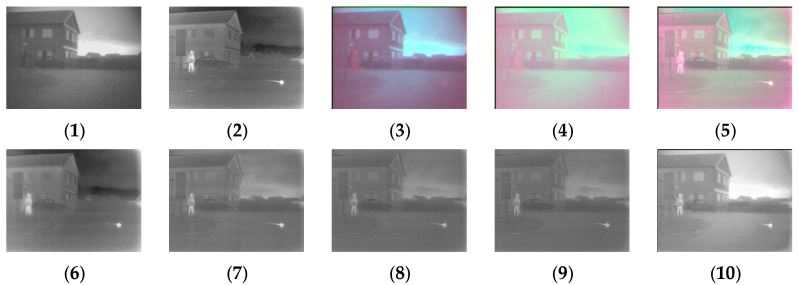
Comparison of experimental results of the sixth set of graphs in the TNO dataset. (**1**) visible image; (**2**) infrared image; (**3**) Li [17]; (**4**) Zhang [18]; (**5**) Wang [23]; (**6**) GTF [24]; (**7**) LatLRR [25]; (**8**) MSVD [26]; (**9**) FPDE [27]; (**10**) Our.

**Figure 10:**
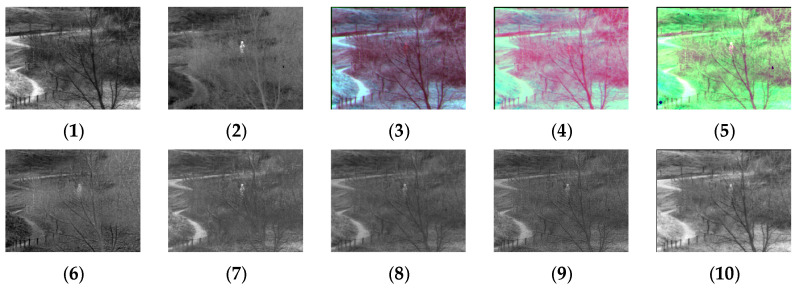
Comparison of experimental results of the seventh set of graphs in the TNO dataset. (**1**) visible image; (**2**) infrared image; (**3**) Li [17]; (**4**) Zhang [18]; (**5**) Wang [23]; (**6**) GTF [24]; (**7**) LatLRR [25]; (**8**) MSVD [26]; (**9**) FPDE [27]; (**10**) Our.

Figure 4, Figure 5, Figure 6, Figure 7, Figure 8, Figure 9 and Figure 10 show the fusion results obtained by the proposed improved algorithm and the corresponding comparative experiment using the source image simulation of seven groups of the TNO dataset. In these results, (1) and (2) are visible and infrared source images, respectively; (3) is the fusion result of Li’s method [17]; (4) is the fusion result of Zhang’s method [18]; (5) is the fusion result of Wang’s method [23]; (6) is the fusion result of GTF’s method [24]; (7) is the fusion result of the LatLRR method [25]; (8) is the fusion result of the MSVD method [26]; (9) is the fusion result of the FPDE method [27]; (10) is the fusion result of the improved method Our.

By observing all the experiments on the TNO dataset, we can find that there is not much difference between Figure 3 and Figure 10 in the surrounding environment, and the key information in the figure can distinguish the rough outline. Combining the comparison of seven groups of images, we can conclude that the results of the fusion method shown in Figure 3 are the most natural color, but the target information is not very prominent. The results of the fusion method shown in Figure 4 and Figure 5 are bright as a whole. As shown in Figure 6, the target information of the fusion method is prominent, but the lack of surrounding environment information in the retained visible image is noticeable, and the overall image is closer to the infrared image. The results of the fusion method shown in Figure 7, Figure 8 and Figure 9 are basically similar, and closer to the visible image than Figure 6. The result graphs of the fusion method shown in Figure 10 not only highlight the target information in the infrared image, but also are closest to the visible image.

#### 4.1.2. MSRS Dataset

**Figure 11:**
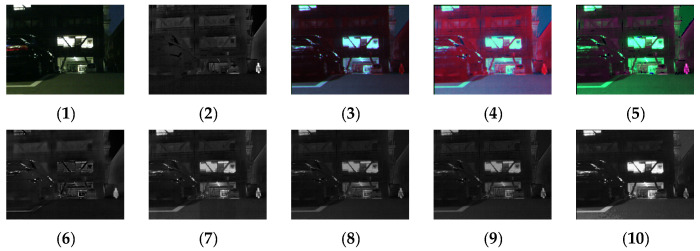
Comparison of experimental results of the first set of graphs in the MSRS dataset. (**1**) visible image; (**2**) infrared image; (**3**) Li [17]; (**4**) Zhang [18]; (**5**) Wang [23]; (**6**) GTF [24]; (**7**) LatLRR [25]; (**8**) MSVD [26]; (**9**) FPDE [27]; (**10**) Our.

**Figure 12:**
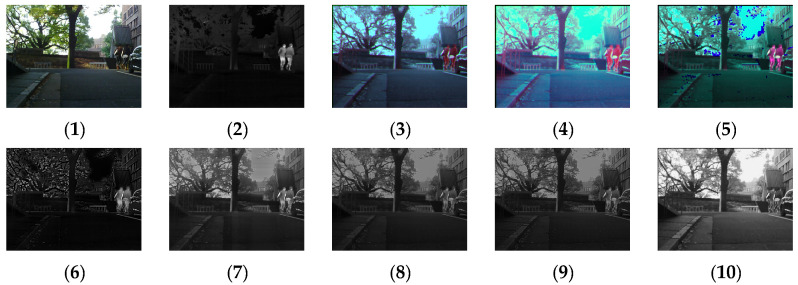
Comparison of experimental results of the second set of graphs in the MSRS dataset. (**1**) visible image; (**2**) infrared image; (**3**) Li [17]; (**4**) Zhang [18]; (**5**) Wang [23]; (**6**) GTF [24]; (**7**) LatLRR [25]; (**8**) MSVD [26]; (**9**) FPDE [27]; (**10**) Our.

**Figure 13:**
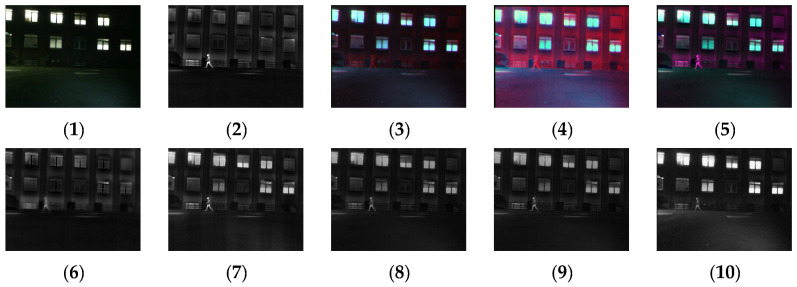
Comparison of experimental results of the third set of graphs in the MSRS dataset. (**1**) visible image; (**2**) infrared image; (**3**) Li [17]; (**4**) Zhang [18]; (**5**) Wang [23]; (**6**) GTF [24]; (**7**) LatLRR [25]; (**8**) MSVD [26]; (**9**) FPDE [27]; (**10**) Our.

**Figure 14:**
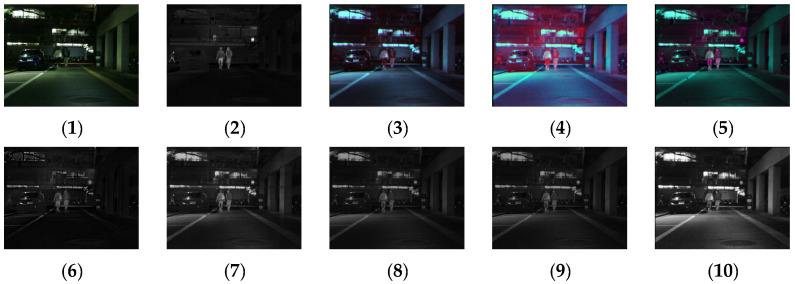
Comparison of experimental results of the fourth set of graphs in the MSRS dataset. (**1**) visible image; (**2**) infrared image; (**3**) Li [17]; (**4**) Zhang [18]; (**5**) Wang [23]; (**6**) GTF [24]; (**7**) LatLRR [25]; (**8**) MSVD [26]; (**9**) FPDE [27]; (**10**) Our.

Figure 11, Figure 12, Figure 13 and Figure 14 show the fusion results obtained by the proposed improved algorithm and the corresponding comparative experiment using the source image simulation of four groups of the MSRS dataset. The images corresponding to (1) to (10) are the same as in the previous section.

By observing all the experiments on the MSRS dataset, we can find that there is not much difference between Figure 3 and Figure 10 in the surrounding environment, and the key information in the figure can distinguish the rough outline. Combining the comparison of seven groups of images, we can conclude that the results of the fusion method shown in Figure 3 are the most natural color, but the target information is not very prominent. As shown in Figure 4, the experimental results are red and bright as a whole. As shown in Figure 5, the target information of the fusion method is prominent, but there are distortion problems in some positions. As shown in Figure 6, the results of the fusion method are darker as a whole, which are closer to the infrared image. The results of the fusion method shown in Figure 7, Figure 8 and Figure 9 are basically similar, and closer to the visible image than Figure 6. The result graphs of the fusion method shown in Figure 10 not only highlight the target information in the infrared image, but also are closest to the visible image.

### 4.2. Objective Evaluation

Above, we used 11 groups of source images for the improved method Our, and conducted experimental comparison with seven algorithms, respectively, and carried out subjective evaluation on the fusion results of the eight algorithms. This section will evaluate the performance of these eight fusion algorithms through three evaluation indexes, such as spatial frequency (SF).

#### 4.2.1. TNO Dataset

**Figure 15:**
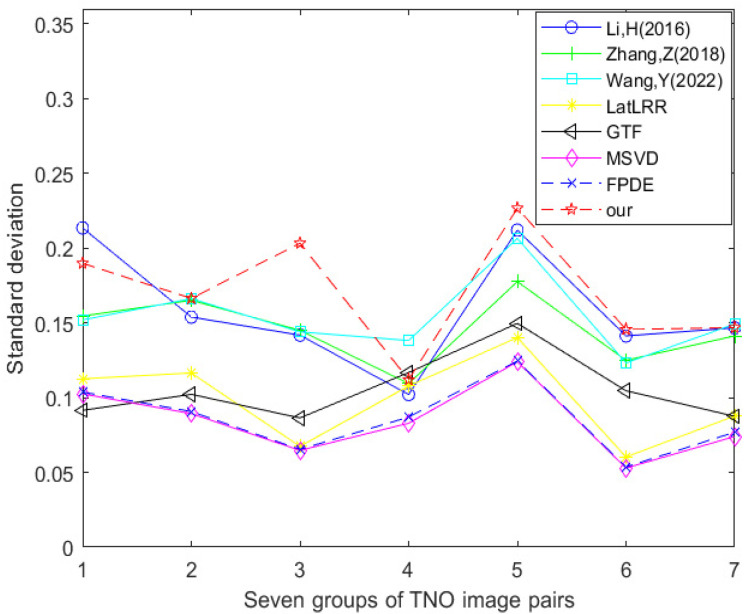
Line chart of standard deviation (TNO dataset) [17,18,23,24,25,26,27].

Table 1 and Figure 15 show the data table and corresponding line graph of the standard deviation evaluation index obtained by experimental comparison of seven other fusion methods and the improved method Our using seven TNO dataset image pairs, respectively. By analyzing the information in the data table and line chart, it can be found that 1/7 of the standard deviation best values are generated using the method proposed by Li et al., 2/7 of the standard deviation best values are generated using the method proposed by Wang et al., and 4/7 of the standard deviation best values are generated using the improved method Our. This shows that in the comparative experiment of these eight fusion methods, the optimal method in terms of standard deviation is the improved method Our.

**Figure 16:**
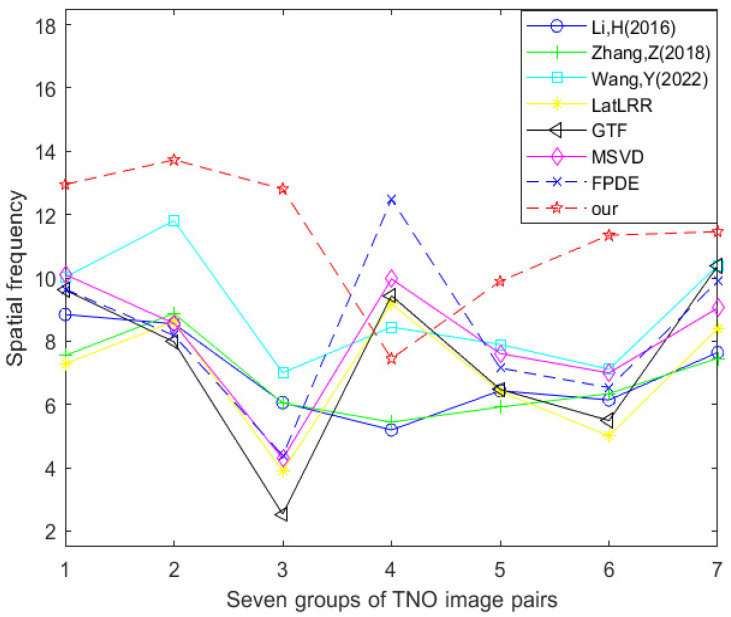
Line chart of spatial frequency (TNO dataset) [17,18,23,24,25,26,27].

Table 2 and Figure 16 show the data table and corresponding line graph of the spatial frequency evaluation index obtained by experimental comparison of seven other fusion methods and the improved method Our using seven TNO dataset image pairs, respectively. By analyzing the information in the data table and line chart, it can be found that 1/7 of the spatial frequency best values are generated using the FPDE method, and 6/7 of the spatial frequency best values are generated using the improved method Our. This shows that in the comparative experiment of these eight fusion methods, the optimal method in terms of spatial frequency is the improved method Our.

**Figure 17:**
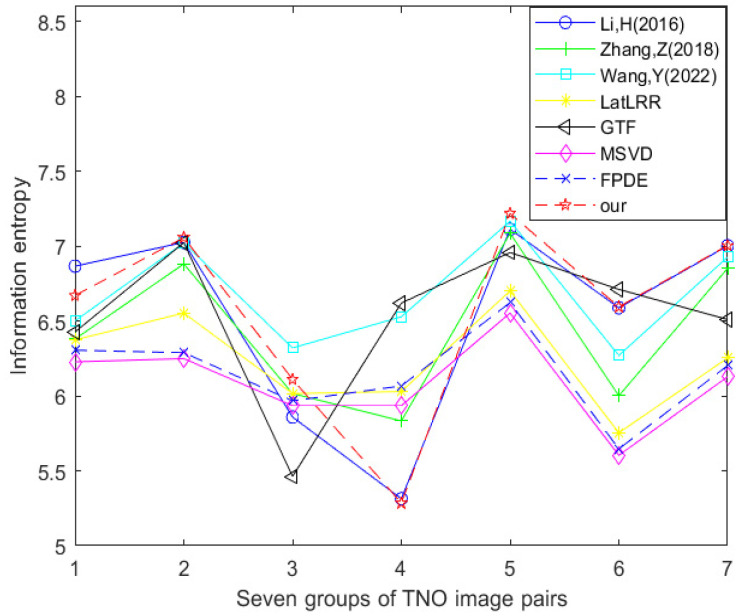
Line chart of information entropy (TNO dataset) [17,18,23,24,25,26,27].

Table 3 and Figure 17 show the data table and corresponding line graph of the information entropy evaluation index obtained by experimental comparison of seven other fusion methods and the improved method Our using seven TNO dataset image pairs, respectively. By analyzing the information in the data table and line chart, it can be found that 1/7 of the information entropy best values are generated using the method proposed by Li et al. [17], 1/7 of the information entropy best values are generated using the GTF method, 2/7 of the information entropy best values are generated using the method proposed by Wang et al. [18], and 3/7 of the information entropy best values are generated using the improved method Our. This shows that in the comparative experiment of these eight fusion methods, the optimal method in terms of information entropy is the improved method Our.

#### 4.2.2. MSRS Dataset

**Figure 18:**
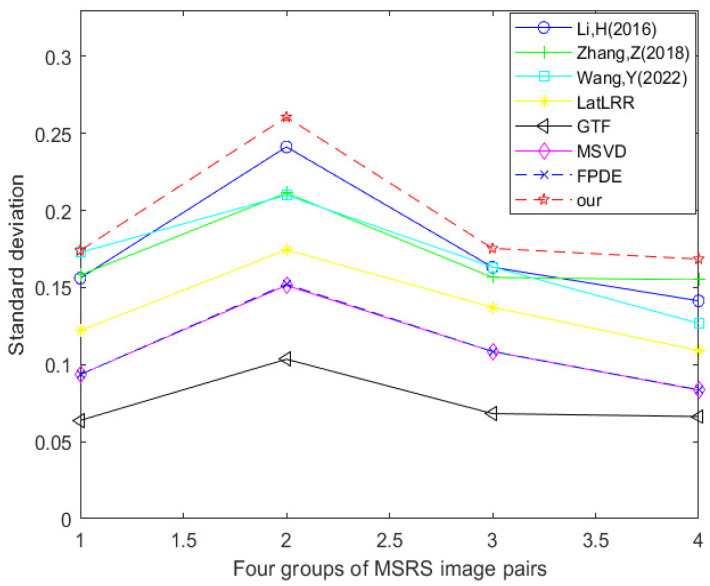
Line chart of standard deviation (MSRS dataset) [17,18,23,24,25,26,27].

Table 4 and Figure 18 show the data table and corresponding line graph of the standard deviation evaluation index obtained by experimental comparison of seven other fusion methods and the improved method Our using four MSRS dataset image pairs. By analyzing the information in the data table and line chart, it can be found that 100% of the standard deviation best values are generated using the improved method Our. This shows that in the comparative experiment of these eight fusion methods, the optimal method in terms of standard deviation is the improved method Our.

**Figure 19:**
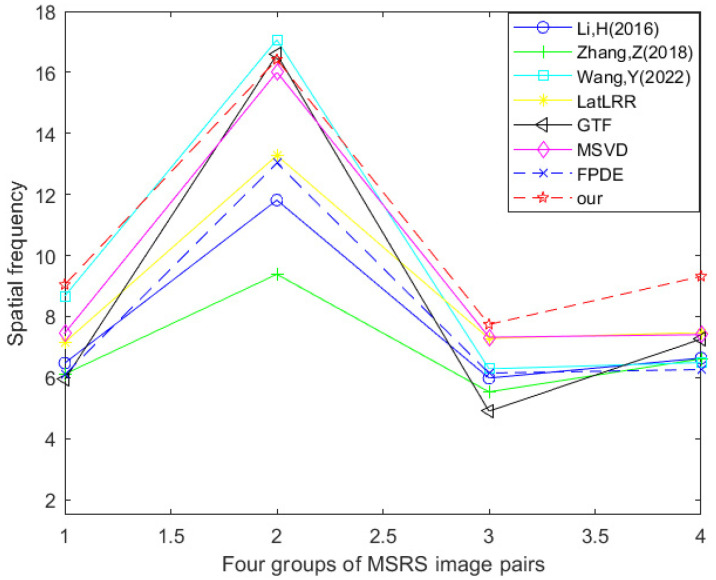
Line chart of spatial frequency (MSRS dataset) [17,18,23,24,25,26,27].

Table 5 and Figure 19 show the data table and corresponding line graph of the spatial frequency evaluation index obtained by experimental comparison of seven other fusion methods and the improved method Our using four MSRS dataset image pairs. By analyzing the information in the data table and line chart, it can be found that 25% of the spatial frequency best values are generated using the method proposed by Wang et al., and 75% of the spatial frequency best values are generated using the improved method Our. This shows that in the comparative experiment of these eight fusion methods, the optimal method in terms of spatial frequency is the improved method Our.

**Figure 20:**
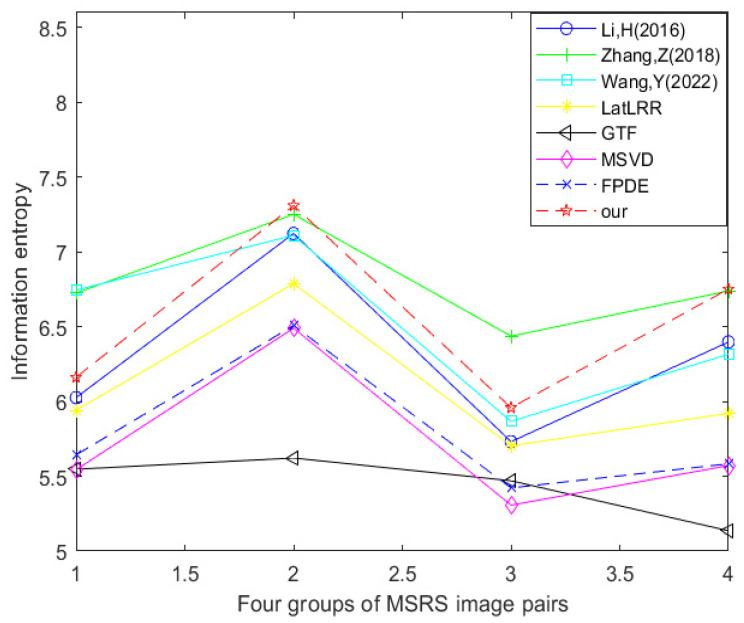
Line chart of information entropy (MSRS dataset) [17,18,23,24,25,26,27].

Table 6 and Figure 20 show the data table and corresponding line graph of the information entropy evaluation index obtained by experimental comparison of seven other fusion methods and the improved method Our using four MSRS dataset image pairs. By analyzing the information in the data table and line chart, it can be found that 25% of the information entropy best values are generated using the method proposed by Zhang et al., 25% of the information entropy best values are generated using the method proposed by Wang et al., and 50% of the information entropy best values are generated using the improved method Our. This shows that in the comparative experiment of these eight fusion methods, the optimal method in terms of information entropy is the improved method Our.

On the whole, most of the optimal values of both TNO dataset and MSRS dataset are concentrated in the method proposed in this paper. Then, the Wilcoxon signed rank test is used to test our proposed method. On the basis of these 11 image pairs, we pair seven comparative methods with the method proposed in this paper on three indicators. First, the null assumption is that the data difference between the two groups is zero; the alternative hypothesis is that there are differences in the data between the two groups. We pair the seven methods with the method proposed in this paper, and since there are three indicators, there are 21 sets of data tests. According to the Wilcoxon signed rank test, our difference value is assumed to be the data index of the method in this paper minus the data index of the comparative method. When the value of the method in this paper is higher than that of the comparative method for an image pair, it is positive rank, and vice versa. Secondly, the difference values are ranked according to their absolute values, and the ranking is assigned in order from small to large in absolute value. Then, we find the sum of the positive ranking and the negative ranking, respectively. Our test statistic W is the smallest absolute value of the sum of positive rankings and the sum of negative rankings. Since we have a total of 11 image pairs, *n* = 11. To determine whether the null hypothesis should be rejected, we refer to the Wilcoxon signed rank test critical value table to find the critical values. The critical value corresponding to a significance level α of 0.1 and *n* = 11 is 13. If our test statistic W is less than or equal to the critical value 13 in the table, we can reject the null hypothesis. Otherwise, we cannot reject the null hypothesis.

(1) According to the calculation, in comparison with the method proposed by Li, we obtain the test statistic W_1_ = 9 using the standard deviation index, the test statistic W_2_ = 0 using the spatial frequency index, and the test statistic W_3_ = 11 using the information entropy index. Since the test statistic W for the three indexes is less than the critical value 13, we reject the null hypothesis, and there is sufficient evidence to prove that there are significant differences between the two methods. Moreover, the sum of our positive rank rankings is much larger than the sum of our negative rank rankings. So, compared with the method proposed by Li, the method proposed in this paper demonstrates a significant improvement in the three indexes. (2) Similarly, in comparison to the method proposed by Zhang, three test statistics are obtained as W_1_ = 0, W_2_ = 0, and W_3_ = 27. We reject the null hypothesis for the index of standard deviation and spatial frequency, while we cannot reject the null hypothesis for the index of information entropy. In other words, compared with the method proposed by Zhang, the method proposed in this paper demonstrates a significant improvement in standard deviation and spatial frequency index. However, for the index of information entropy, there is almost no difference with the method proposed by Zhang. (3) In comparison with the method proposed by Wang, three test statistics are obtained as W_1_ = 10, W_2_ = 5, and W_3_ = 28. We reject the null hypothesis for the index of standard deviation and spatial frequency, while we cannot reject the null hypothesis for the index of information entropy. In other words, compared with the method proposed by Wang, the method proposed in this paper demonstrates a significant improvement in standard deviation and spatial frequency index. However, for the index of information entropy, there is almost no difference with the method proposed by Wang. (4) Compared with the LatLRR method, three test statistics are obtained as W_1_ = 0, W_2_ = 2, and W_3_ = 8. We reject the null hypothesis for all three indexes. In other words, compared with the LatLRR method, the method proposed in this paper demonstrates a significant improvement in the three indexes. (5) Compared with the GTF method, three test statistics are obtained as W_1_ = 1, W_2_ = 4, and W_3_ = 11, and we reject the null hypothesis for all three indexes. In other words, compared with the GTF method, the method proposed in this paper demonstrates a significant improvement in the three indexes. (6) Compared with the MSVD method, three test statistics are obtained as W_1_ = 0, W_2_ = 7, and W_3_ = 4. We reject the null hypothesis for all three indexes. In other words, compared with the MSVD method, the method proposed in this paper has a significant improvement in the three indexes. (7) Compared with the FPDE method, three test statistics are obtained as W_1_ = 0, W_2_ = 9, and W_3_ = 7. We reject the null hypothesis for all three indexes. In other words, compared with the FPDE method, the method proposed in this paper demonstrates a significant improvement in the three indexes.

To sum up, in the statistical test compared with the seven comparative methods, the method proposed in this paper shows significant improvement in other indicators except that there is almost no difference between the method proposed by Zhang and the method proposed by Wang in the index of information entropy. Therefore, the improved algorithm proposed in this paper is superior to the other seven comparative methods.

## 5. Conclusions

Based on the characteristics of six dual-mode cells of rattlesnakes and the standard mathematical model of pulse coupled neural networks, we proposed a new rattlesnake fusion method. Compared with the image fusion method based on deep learning, it does not need to obtain a significant amount of training data. The overall brightness of the fusion image obtained by our method is moderate, the target information is prominent, and the detail information is rich. And the experimental data compared with other fusion methods prove that the proposed method demonstrates good performance.

## Figures and Tables

**Table 1:** Standard deviation (TNO).

	Li [17]	Zhang [18]	Wang [23]	LatLRR [24]	GTF [25]	MSVD [26]	FPDE [27]	Our
Picture group 1	**0.2135**	0.1546	0.1520	0.1126	0.0915	0.1025	0.1037	0.1898
Picture group 2	0.1539	0.1651	0.1662	0.1165	0.1022	0.0893	0.0907	**0.1664**
Picture group 3	0.1418	0.1450	0.1440	0.0674	0.0864	0.0647	0.0654	**0.2033**
Picture group 4	0.1021	0.1094	**0.1382**	0.1078	0.1168	0.0829	0.0870	0.1120
Picture group 5	0.2120	0.1776	0.2065	0.1402	0.1497	0.1240	0.1245	**0.2267**
Picture group 6	0.1413	0.1250	0.1232	0.0603	0.1046	0.0529	0.0536	**0.1456**
Picture group 7	0.1467	0.1413	**0.1493**	0.0877	0.0875	0.0740	0.0769	0.1468

**Table 2:** Spatial frequency (TNO).

	Li [17]	Zhang [18]	Wang [23]	LatLRR [24]	GTF [25]	MSVD [26]	FPDE [27]	Our
Picture group 1	8.8415	7.5451	10.0236	7.2846	9.6134	10.1040	9.6411	**12.9517**
Picture group 2	8.5481	8.8754	11.8046	8.6205	7.9767	8.5605	8.1801	**13.7281**
Picture group 3	6.0567	6.0392	6.9992	3.8864	2.5295	4.2855	4.3835	**12.8079**
Picture group 4	5.1933	5.4416	8.4458	9.1842	9.4375	9.9641	**12.4838**	7.4459
Picture group 5	6.4334	5.9170	7.8947	6.4058	6.4738	7.6088	7.1472	**9.8928**
Picture group 6	6.1416	6.3427	7.1119	5.0020	5.4890	6.9946	6.5330	**11.3467**
Picture group 7	7.6393	7.4567	10.3869	8.3988	10.3762	9.0481	9.8858	**11.4572**

**Table 3:** Information entropy (TNO).

	Li [17]	Zhang [18]	Wang [23]	LatLRR [24]	GTF [25]	MSVD [26]	FPDE [27]	Our
Picture group 1	**6.8670**	6.3806	6.5015	6.3750	6.4274	6.2278	6.3057	6.6705
Picture group 2	7.0262	6.8819	7.0225	6.5537	7.0241	6.2495	6.2887	**7.0583**
Picture group 3	5.8593	6.0163	**6.3209**	6.0161	5.4626	5.9345	5.9674	6.1107
Picture group 4	5.3138	5.8331	**6.5289**	6.0297	6.6205	5.9350	6.0671	5.2849
Picture group 5	7.1138	7.0863	7.1669	6.7016	6.9581	6.5541	6.6235	**7.2189**
Picture group 6	6.5886	6.0013	6.2707	5.7537	**6.7120**	5.6038	5.6463	6.5948
Picture group 7	7.0030	6.8522	6.9275	6.2539	6.5104	6.1284	6.2031	**7.0052**

**Table 4:** Standard deviation (MSRS).

	Li [17]	Zhang [18]	Wang [23]	LatLRR [24]	GTF [25]	MSVD [26]	FPDE [27]	Our
Picture group 1	0.1561	0.1581	0.1728	0.1220	0.0638	0.0936	0.0935	**0.1740**
Picture group 2	0.2413	0.2114	0.2099	0.1745	0.1035	0.1514	0.1522	**0.2606**
Picture group 3	0.1632	0.1566	0.1634	0.1372	0.0682	0.1085	0.1085	**0.1754**
Picture group 4	0.1414	0.1550	0.1268	0.1092	0.0663	0.0835	0.0837	**0.1685**

**Table 5:** Spatial frequency (MSRS).

	Li [17]	Zhang [18]	Wang [23]	LatLRR [24]	GTF [25]	MSVD [26]	FPDE [27]	Our
Picture group 1	6.4717	6.1116	8.6695	7.1532	5.9345	7.4550	6.0949	**9.0415**
Picture group 2	11.8137	9.3834	**17.0645**	13.2754	16.5825	16.0087	13.0278	16.4181
Picture group 3	5.9809	5.5280	6.2842	7.2816	4.8935	7.3191	6.1444	**7.7393**
Picture group 4	6.6357	6.6135	6.4892	7.4747	7.2597	7.4051	6.2608	**9.3136**

**Table 6:** Information entropy (MSRS).

	Li [17]	Zhang [18]	Wang [23]	LatLRR [24]	GTF [25]	MSVD [26]	FPDE [27]	Our
Picture group 1	6.0258	6.7251	**6.7449**	5.9413	5.5473	5.5442	5.6435	6.1622
Picture group 2	7.1236	7.2519	7.1105	6.7882	5.6220	6.4905	6.5077	**7.3097**
Picture group 3	5.7315	**6.4371**	5.8671	5.7037	5.4701	5.3070	5.4223	5.9582
Picture group 4	6.3980	6.7393	6.3160	5.9207	5.1357	5.5716	5.5835	**6.7519**

## Data Availability

The data in this study are available on request to the first author.
